# Knowledge of confidentiality, consent and information disclosure is not affected by position or experience in adult critical care

**DOI:** 10.1186/cc12431

**Published:** 2013-03-19

**Authors:** M Lowings, A Molokhia, C Anderson, S Cowman, T Dixson, K El-Boghdadly, L Williams

**Affiliations:** 1 St Thomas' Hospital, London, UK; 2 Lewisham University NHS Trust, London, UK; 3 King's College Hospital, London, UK; 4 Great Ormond Street Hospital, London, UK; 5 Queen Elizabeth Hospital, Woolwich, UK; 6 Princess Royal University Hospital, Bromley, UK

## Introduction

Teaching of medical ethical issues including confidentiality and consent have long been a small part of the medical curriculum. These issues are more complex in an ICU where patients may lack capacity. Documents such as Good Medical Practice 1995, Confidentiality 2009 and the Mental Capacity Act 2005 give guidance to medical professionals in these matters in the UK.

## Methods

A questionnaire was distributed amongst staff in four ICUs in South London. Results were analysed according to level of experience and background (medical/nursing or allied health professional (AHP)).

## Results

Of 225 questionnaires distributed, the response rate was 66% (31% doctors, 56% nurses and 13% AHP). Staff with either less than 1 year experience or greater than 10 years experience had the greatest exposure to the Mental Capacity Act and Data Protection Act, suggesting a gap in knowledge in staff with intermediate experience. Knowledge of the Caldicott principles were unaffected by experience, with many experienced respondents having 'No Idea'. The majority of respondents (unaffected by experience) felt that when giving information to relatives face to face, relatives should be kept fully informed. When giving information over the telephone, most doctors felt the response should be tailored to the knowledge of the person being spoken to whilst nurses were split between tailoring the response, giving full information, setting up a password system and not giving any information at all. Most respondents felt date of birth and hospital number constituted 'Patient Identifiable Information'. However, experienced staff did not appreciate the importance of unusual diagnosis and clinical photographs as also being able to identify patients. Similarly, the majority knew that the patient themselves identified the 'Next of Kin' but 7% (unaffected by experience) felt this was decided by the family and felt the family could decide on resuscitation status. When consent is required for an elective procedure in a patient who lacks capacity, doctors tended to have a better understanding of the need to delay the procedure where possible than nurses, the majority of which felt this could be decided by the next of kin or two consultant doctors. Most doctors felt that 'Acting in the Patient's Best Interests' would mean doing what would give the patient the best outcome rather than doing what the patient would have wanted (unaffected by experience). The majority of staff, on answering this questionnaire, felt that they lacked sufficient knowledge on the subject and most felt annual reminders would be useful.

## Conclusion

The ICU is an environment where issues of consent, confidentiality and disclosure of information occur daily. Staff feel they lack knowledge in these areas that is unaffected by their experience. We need to ensure that all staff have the necessary knowledge to deal with these situations.

